# Role of Scrib and Dlg in anterior-posterior patterning of the follicular epithelium during *Drosophila *oogenesis

**DOI:** 10.1186/1471-213X-9-60

**Published:** 2009-12-01

**Authors:** Qi Li, Ling Shen, Tianchi Xin, Wenjuan Xiang, Wenlian Chen, Yin Gao, Mingwei Zhu, Lingzhu Yu, Mingfa Li

**Affiliations:** 1 MoE Key Laboratory of Developmental Genetics and Neuropsychiatric Diseases, Bio-X Center, School of Life Science and Biotechnology, Shanghai Jiao Tong University, 200240 Shanghai, PR China

## Abstract

**Background:**

Proper patterning of the follicle cell epithelium over the egg chamber is essential for the *Drosophila *egg development. Differentiation of the epithelium into several distinct cell types along the anterior-posterior axis requires coordinated activities of multiple signaling pathways. Previously, we reported that *lethal(2)giant larvae *(*lgl*), a *Drosophila *tumor suppressor gene, is required in the follicle cells for the posterior follicle cell (PFC) fate induction at mid-oogenesis. Here we explore the role of another two tumor suppressor genes, *scribble *(*scrib*) and *discs large *(*dlg*), in the epithelial patterning.

**Results:**

We found that removal of *scrib *or *dlg *function from the follicle cells at posterior terminal of the egg chamber causes a complete loss of the PFC fate. Aberrant specification and differentiation of the PFCs in the mosaic clones can be ascribed to defects in coordinated activation of the EGFR, JAK and Notch signaling pathways in the multilayered cells. Meanwhile, the clonal analysis revealed that loss-of-function mutations in *scrib/dlg *at the anterior domains result in a partially penetrant phenotype of defective induction of the stretched and centripetal cell fate, whereas specification of the border cell fate can still occur in the most anterior region of the mutant clones. Further, we showed that *scrib *genetically interacts with *dlg *in regulating posterior patterning of the epithelium.

**Conclusion:**

In this study we provide evidence that *scrib *and *dlg *function differentially in anterior and posterior patterning of the follicular epithelium at oogenesis. Further genetic analysis indicates that *scrib *and *dlg *act in a common pathway to regulate PFC fate induction. This study may open another window for elucidating role of *scrib/dlg *in controlling epithelial polarity and cell proliferation during development.

## Background

The follicle cell (FC) epithelium over the egg chamber in *Drosophila *ovary plays a pivotal role in the egg development. At oogenesis, follicular epithelial cells along the anterior-posterior (AP) axis are specified and differentiated into several distinct cell types that will subsequently either undergo a series of morphogenetic changes, or extend the germline-soma interactions [1-3]. While most of the epithelial FC subpopulations contribute to construction of the eggshell along with its specialized structures such as dorsal appendages through complex morphogenesis, the specified PFCs initiate establishment of the oocyte polarity, and determine the AP and dorsal-ventral (DV) axes of the resulting embryos. Thus, patterning of the follicular epithelium is an essential step for the proper development of the egg.

*Drosophila *oogenesis begins with formation of the 16-cell germline cyst in the germarium of the ovaries that is composed of 15 nurse cells and one oocyte. Each germline cyst is then encapsulated by a monolayer of the somatic stem cell-derived FCs [1,4]. After the encapsulated cysts leave the germarium, the surrounding somatic FCs develop into a sheet of cuboidal epithelial cells and a pair of polar cells at each pole of the nascent egg chamber. As oogenesis proceeds, the follicular epithelium in the egg chamber becomes progressively polarized along the AP axis [5]. Early on, two terminal domains are differentiated from the mainbody region in the epithelial FC layer. Further, cells in each terminal domain can adopt three different terminal fates, depending on their locations away from the poles of the chamber [1,6,7]. In this way, the mirror image prepattern of the terminal domains in the epithelium is generated. This symmetry was broken at mid-oogenesis when Gurken signal, produced in the oocyte, activates the EGFR signaling pathway in the surrounding FCs at the posterior of the egg chambers, defining a posterior fate in those cells [5,8-11]. At this time, AP patterning of the epithelium is established. Starting from stage 7, the epithelial FCs cease proliferation and enter an endocycle [12,13]. By stage 8, all specified FC types in the epithelium along the AP axis are differentiated into five distinct subpopulations, known as border, stretched, centripetal, main body and posterior cells.

Once becoming subdivided into various cell types along the AP axis, the epithelial FCs undergo dramatic cell shape change and directed migration at middle and late oogenesis [1,2]. These morphogenetic processes include: (1) During stage 9, a group of 6-10 border cells delaminate from the anterior tip of the epithelium and migrate between the nurse cells to the anterior end of the oocyte; (2) At the same time, the majority of the FCs, including the centripetal, mainbody and PFC cells, move posteriorly to form a columnar epithelium covering the growing oocyte, while the stretched cells adjacent to the border cells flatten to be in association with the nurse cells as a squamous epithelium; (3) At stage 10b, the centripetal cells migrate between the nurse cells and the oocyte towards the center of the egg chamber to cover the anterior region of the oocyte. The unique morphogenesis above conferred by the patterned follicular epithelium is destined to bring the vast majority of the FCs into contact with the oocyte for ultimate formation of the eggshell with its specialized structures.

Apart from being involved in the deposition of a functional eggshell, the specified PFCs function in the axial patterning of the oocyte and the resultant embryo. During stage 6-7, the PFCs signal back to the oocyte, causing the reorganization of its microtubule (MT) cytoskeleton [14,15]. This directs the MT-dependent localization of *bicoid *and *oskar *mRNA to the anterior and posterior pole of the oocyte respectively, thus defining the AP polarity of the oocyte, and the resulting embryo's AP axis [15-17]. The repolarization of the MT cytoskeleton also triggers the migration of the nucleus from the posterior to the dorsal anterior corner in the oocyte [18]. At this time, the localized Grk around the nucleus signals for a second time to induce the overlying main body cells to adopt a dorsal, rather than a ventral fate, specifying the pattern of the DV axis [10,11,19-21]. Thus, the PFCs execute dual functions for the egg development.

Each cell in a given tissue or organ can interpret its positional information provided by signaling molecules from a local source to adopt a specific fate. Several lines of evidence have indicated that the JAK/STAT and EGFR pathways, elicited by the signaling ligands Unpaired from the polar cells and Gurken from the oocyte respectively, function in the AP patterning of the entire follicular epithelium. Prior to stage 6 of oogenesis, the graded JAK/STAT pathway activities induce the division of the FC layer into the two terminal domains and the main body domain, and three default anterior fates within each terminal [6,7,22]. Later on, activation of EGFR signaling pathway in the posterior terminal instructs those terminal cells to adopt a posterior, rather than an anterior fate. Meanwhile, the Notch signaling activity is necessary for differentiation of those specified FC types by controlling a switch from mitosis to endocycle in the epithelium at early mid-oogenesis [12,13]. Although essential role of the JAK signaling in patterning the AP axis of the follicular epithelium is established, current data also suggest that other signals present at the terminal are required for specification of the distinct anterior fates, particularly induction of the stretched and centripetal cell subpopulations[1,6,7].

To date, several genes have been showed to regulate the above three well-characterized signalling pathways at oogenesis, thereby being implicated in the follicular patterning [23-28]. For example, the Hippo (Hpo) tumor suppressor pathway components are involved in the PFC fate induction through modulating the Notch activity [24-26,29]. Recently, we reported that *lgl *is required in the FCs for specification and differentiation of the PFCs at mid-oogenesis [30]. In this study we tested another two *Drosophila *tumor suppressor genes *scrib *and *dlg *for their role in PFC fate induction. Removal of *scrib *or *dlg *function from the epithelial FCs at the posterior resulted in a loss of the PFC fate, subsequently causing oocyte polarity defects. Further, we showed that the aberrant coordinated activities of EGFR, JAK/STAT and Notch pathways elicited by mutations in *scrib/dlg *are causally linked to defective specification of the PFC fate in the mosaic clones. We also investigated whether *scrib/dlg *are implicated in anterior patterning of the follicular epithelium. Inactivation of *scrib *or *dlg *in the FCs at the anterior disrupted specification of the stretched and centripetal cell fate, whereas differentiation of the border cells can still occur in the most anterior regions of the mutant clones. Finally, we provided genetic evidence that *scrib *and *dlg *function in posterior patterning of the epithelium in a common pathway.

## Results

### *scrib/dlg *are required for specification and differentiation of the PFCs

**Figure 1:**
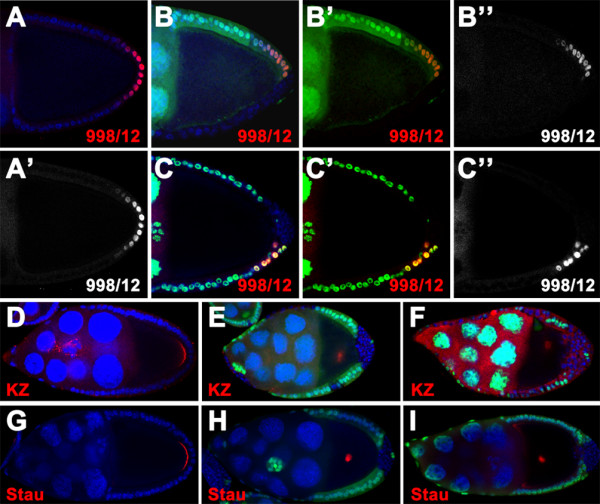
**Loss of *scrib or dlg *function causes defects in specification and differentiation of the PFCs**. Wild-type (A, D and G) and mutant egg chambers containing *scrib*^2 ^(B, E and H) or *dlg*^*m*52 ^(C, F and I) clones at the posterior marked by the absence of nuclear GFP (green in B, C, E, F and H) or β-gal (green in I), stained for nuclei (DAPI, blue) and β-gal(red in A-F) or Stau (red in G-I). **(A-C) **Expression of the PFC marker 998/12 can be observed in stage 10 wild type egg chambers (A, A'), whereas loss of 998/12 expression is evident in the *scrib*^2 ^(B-B") and *dlg*^*m*52 ^(C-C") clone cells. Note that 998/12 is still present in the remaining wild-type posterior cells (B, C), indicating that *scrib *and *dlg *act cell-autonomously in specifying the PFCs. **(D-F) **In the wild type, Kinesin-lacZ is localized at the posterior of the oocyte at stage 9 chambers (D). But the fusion protein is mislocalized to the center when the FCs at the posterior are homozygous for *scrib*^2 ^(E) and *dlg*^*m*52 ^(F). **(G-I) **In stage 10 wild type egg chambers, Stau accumulates at the posterior of the oocyte (G). Stau is mislocalized to the center as a dot when *scrib *(H) or *dlg *(I) is inactivated in FCs at the posterior.

In *Drosophila*, *lgl*, *scrib *and *dlg *are classified as the neoplastic tumor suppressor genes (nTSGs), which work in concert to control the cell polarity and proliferation in epithelial tissues [31-35]. Recently, we have shown that removal of *lgl *function from the FCs at the posterior of egg chambers results in failure of the oocyte to be polarized along AP and DV axis at mid-oogenesis. These defects have been attributed to lacking of the specified PFC fate in the mutant chambers [30]. Here we tested whether other two nTSGs *scrib *and *dlg *are implicated in specification and differentiation of the PFCs. For this purpose, we analyzed the expression of the specific PFC fate marker 998/12 in *scrib *or *dlg *mutant clones. Remarkably, in the stage 9/10 mutant egg chambers, expression of the 998/12 reporter was disrupted in all examined clones at the posterior that are homozygous for the null alleles *scrib*^2 ^or *dlg*^*m*52 ^in a cell-autonomous manner (*scrib*^2^, n = 64; *dlg*^*m*52^, n = 45) (Fig 1B, C). Since proper differentiation of the PFCs is essential for reorganization of the microtubule cytoskeleton in the oocyte, hence for the oocyte polarity formation, it is deducible that mutations in *scrib/dlg *can cause defective microtubule polarization and oocyte polarity at mid-oogenesis. Indeed, both Kinesin-lacZ (KZ) and Staufen were either mislocalized from the posterior pole to the center of the oocyte, or diffused around the cortex of the oocyte at stage 9/10 of oogenesis when *scrib *or *dlg *mutant clones harbored the entire posterior FCs (KZ: *scrib*^2^, 100%, n = 36; *dlg*^*m*52^, 100%, n = 51; Staufen: *scrib*^2^, 100%, n = 57; *scrib*^1^, 100%, n = 66; *dlg*^*m*52^, 100%, n = 50) (Fig 1E, F, H, I and data not shown). Taken together, these results indicated that like *lgl*, *scrib/dlg *are required in FCs in a cell-autonomous manner for specification and differentiation of the PFCs.

### Disruption of the signaling pathways may underlie the defective PFC fate induction in *scrib/dlg *mutants

**Figure 2:**
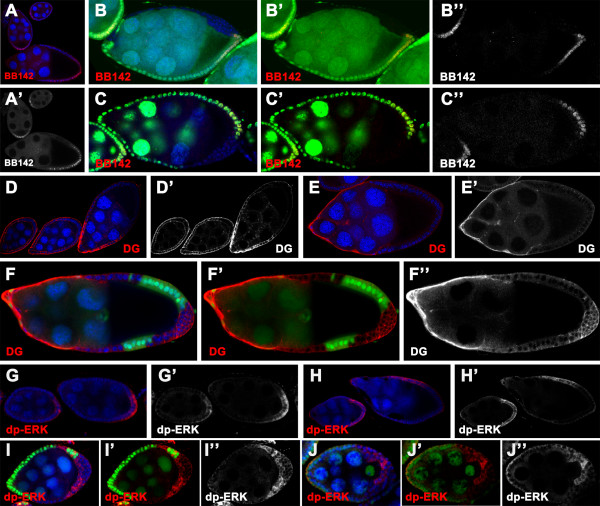
**Loss of *scrib/dlg *function in FCs at the posterior disrupts the EGFR signaling**. Wild type (A, D, E, G and H) and mosaic egg chambers with *scrib*^2 ^(B, F and I) or *dlg*^*m*52 ^(C and J) clones at the posterior, marked by the absence of nuclear GFP (green in B, C, F and I) or β-gal (green in J), stained for nuclei (DAPI, blue), β-gal (red in A-C), DG (red in D-F) or dp-ERK (red in G-J). **(A-C) **In the wild type, expression of *kek *enhancer trap marker BB142 can be observed in the PFCs at stage 6-8 egg chambers (A). Expression of BB142 is completely absent in the *scrib*^2 ^(B-B") and *dlg*^*m*52 ^(C-C") clone cells at stage 8 egg chamber. **(D-F) **In the wild type, DG is evenly expressed in all FCs before stage 6/7, when DG is down regulated in the PFCs (D, D'). At stage 9/10, DG expression is dramatically reduced in all FCs except the AFCs (E, E'). Remarkably, ectopic expression of DG in all cell-membrane domain is evident in the *scrib*^2 ^multilayered clone cells at stage 9/10 chambers (F-F"). **(G-J) **In the wild type, dp-ERK can be detected in the posterior FCs from stage 6-8 (G, G', H and H'), and in dorsal FCs at stage 9 (H, H'). ERK activation can still occur in *scrib*^2 ^(I-I") or *dlg*^*m*52 ^(J-J") mutant posterior FCs at stage 6 egg chamber.

It is known that combinatorial activities of the JAK/STAT, EGFR and Notch signaling pathway determine specification and differentiation of the PFCs [3,6,8,9,12,13,36]. Perturbing one or more of the three pathways may lead to a loss of the PFC fate. We, therefore, examined whether disruption of the above signaling pathways links *scrib/dlg *mutations to the defective PFC fate induction by analyzing the expression of pathway-regulated targets and/or effectors in the mosaic egg chambers. We first chose to test whether loss of the PFC fate in *scrib/dlg *mutant chambers is causally linked to defective EGFR pathway. A enhancer trap line BB142 in which *lacZ *is expressed under the control of *kekkon *(*kek*), a primary downstream target gene of the EGFR pathway, was employed to assay EGFR signaling activities [23,37]. In the wild type (Fig 2A and [23,37]), *kek *expression is detected in the PFCs in stage 7/8 egg chambers, and subsequently becomes restricted to the dorsal FCs at stage 10. Remarkably, loss of *kek *expression is evident in all tested egg chambers at stage 8 with a FRT clone homozygous for *scrib*^2 ^or *dlg*^*m*52 ^at the posterior (*scrib*^2^, n = 62; *dlg*^*m*52^, n = 35) (Fig 2B, C). In the mutant clones only harboring part of the posterior domain, we observed that activation of the EGFR signaling can still occur in the remaining wild type cells (Fig 2B, C), indicating that the EGFR pathway was disrupted in a cell-autonomous fashion. To corroborate this finding, we performed the immuno-staining of the ovaries using the antibody against Dystroglycan (DG), an extracellular matrix receptor that is down-regulated in the PFCs by EGFR signaling starting from stage 6/7 (Fig 2D, E) [38]. Consistently, *scrib/dlg *mutant clones at the posterior of stage 9/10 chambers displayed a cell-autonomous up-regulation of DG expression (*scrib*^2^, 59.1%, n = 22; *scrib*^1^, 60%, n = 10; *dlg*^*m*52^, 38.1%, n = 21) (Fig 2F and data not shown), providing more evidence that loss of *scrib/dlg *function perturbs activation of EGFR signaling at the posterior. Altogether, the results support a notion that disruption of the EGFR signaling is the cause of defective PFC fate induction in *scrib/dlg *mutants.

To better understand how the EGFR signaling is disrupted in the mutant FCs at the posterior, we further examined whether mutations in *scrib/dlg *block activation of ERK, the key signal transducer of EGFR pathway [39,40]. Our analysis revealed that ERK activation can still occur in the posterior *scrib *or *dlg *mutant FCs at stage 6-8 of oogenesis, as indicated in the presence of di-phosphorylated form of ERK (*scrib*^2^, n = 23; *dlg*^*m*52^, n = 28) (Fig 2I, J). These data suggest that disruption of the EGFR signaling pathway at the posterior domains induced by loss of *scrib *or *dlg *may take place downstream of ERK activation.

**Figure 3:**
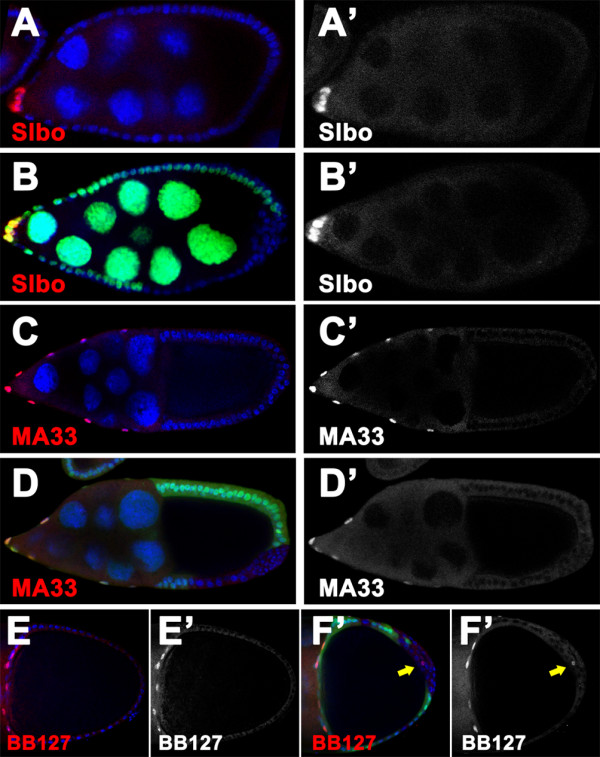
**The *scrib/dlg *mutant FCs at the posterior rarely adopt a default AFC fate**. Wild type (A, C and E) and *dlg*^*m*52 ^(B) or *scrib*^2 ^(D and F) mosaic chambers stained for nuclei (DAPI, blue) and Slbo (red in A and B) or β-gal (red in C-F). The mutant clones are marked by lack of the nuclear GFP (green). **(A, B) **In stage 8 wild type egg chamber Slbo is exclusively expressed in the border cells at the anterior pole (A, A'). This border cell marker is not ectopically expressed in the *dlg*^*m*52 ^clone cells at the posterior (B, B'). **(C, D) **In the wild type, MA33 specifically labels the stretched cells, which cover the nurse cells at the anterior of the stage 10 egg chamber (C, C'). In the mosaic egg chamber, expression of this enhancer trap marker is present in the wild type stretched cells, but absent in *scrib*^2 ^FCs at the posterior (D, D') as the same as the wild type. **(E, F) **The enhancer trap insertion BB127 is specifically expressed in the centripetal cells in stage 10b wild type egg chamber (E, E'), and occasionally labels the *scrib*^2 ^follicle cells at the posterior (F, F'). Note that only one or two mutant cells adopt BB127- expressing cell fate (arrows in F, F').

It has been reported that the posterior FCs with aberrant EGFR signaling adopt a default anterior fate, instead of the PFC fate [8,9,38]. This prompted us to test if it is the case in the *scrib/dlg *mutants by examining expression of a series of the anterior follicle cell (AFC) fate markers. First, we did not detect any expression of either border cell or stretched cell markers in the PFC mutant for *scrib *or *dlg*, as evident in the staining with antibody against Slbo (*scrib*^2^, n = 66; *scrib*^1^, n = 32; *dlg*^*m*52^, n = 54) (Fig 3B and data not shown) (Table 1) or β-gal for MA33 enhancer trap line (*scrib*^2^, n = 37; *dlg*^*m*52^, n = 12) (Fig 3D and data not shown) (Table 1). Further, we analyzed expression pattern of more AFC fate markers in the mosaic chambers using enhancer trap lines BB127, *dpp-lacZ *and L53b, which label the centripetal cells or all AFCs respectively [8,41,42]. As shown in Fig 3F and Table 1, only a small percentage of the mutant clones expressed BB127, *dpp-lacZ *or L53b, indicating that the mutant FCs at the posterior rarely adopt the AFC fate. In this case, loss of *scrib *or *dlg *differs from mutations of the EGFR pathway components in that EGFR pathway mutant FCs at the posterior can routinely adopt a default anterior fate. Overall, the posterior FCs lacking *scrib *or *dlg *do not adopt either the PFC or a default terminal cell fate at mid-oogenesis.

**Table 1:** Expression of the AFC markers in *scrib/dlg *mutant clone cells at the posterior terminal

		Presence of The AFC Fate in PFC Clones	
		
AFC Markers	Cell Types	*scrib* ^2^	*dlg* ^*m*52^
Slbo	Border Cell	0% (n = 66)	0%(n = 54)

MA33	Stretched Cell	0% (n = 37)	0%(n = 12)

BB127	Centripetal Cell	10%(n = 20)	ND

L53b	Anterior Follicle cell	ND	8.3%(n = 36)

*dpp-lacZ*	Anterior Follicle cell	9.1%(n = 22)	ND

**Figure 4:**
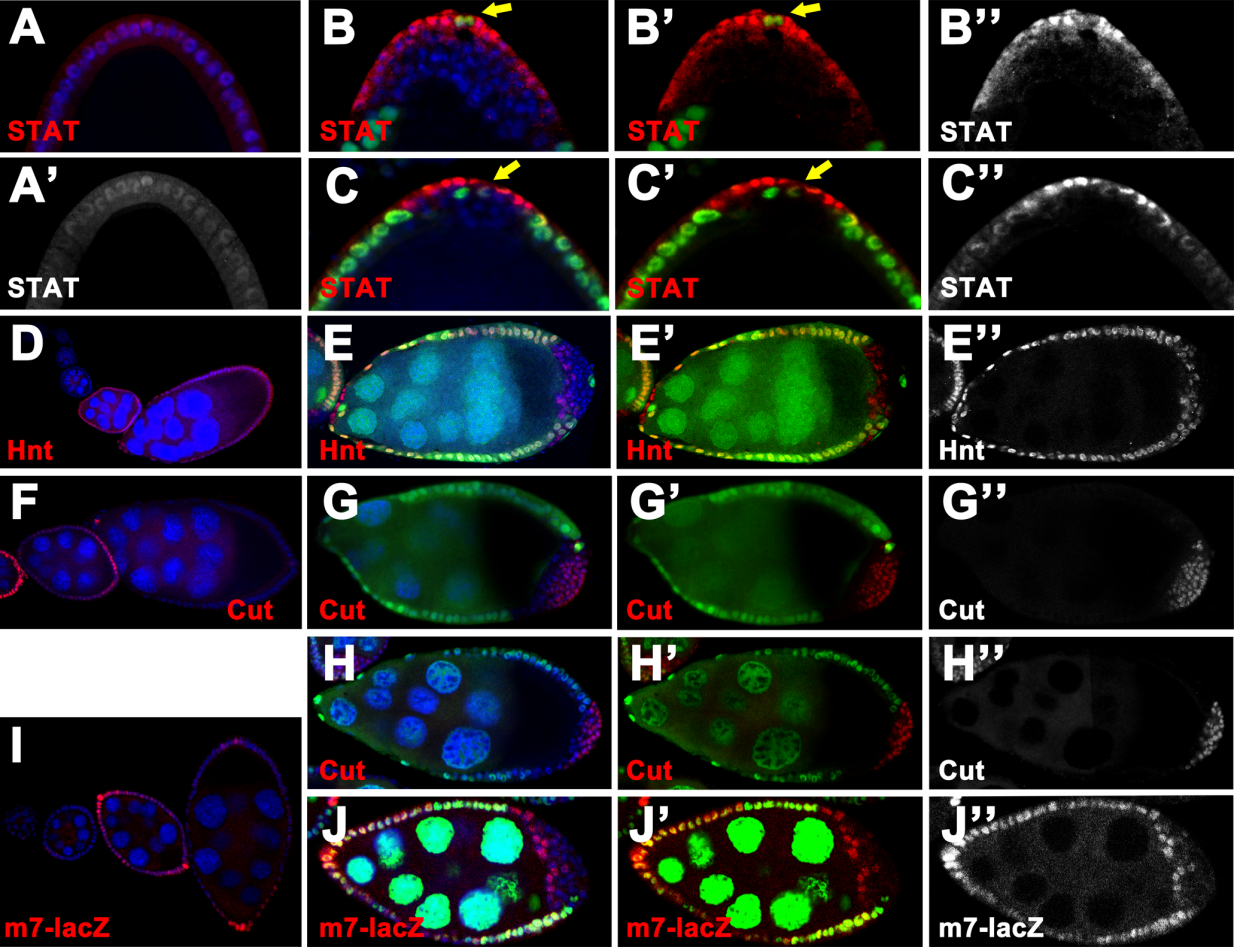
**JAK and Notch signaling can not be coordinately activated in *scrib*/*dlg *mutant FCs at the posterior**. Wild type (A, D, F and I) and mosaic egg chambers with *scrib*^2 ^(B, E and G) or *dlg*^*m*52 ^(C, H and J) clones marked by the absence of nuclear GFP (green in B, E, G and J) or β-gal (green in C and H), stained for nuclei (DAPI, blue) and STAT92E (red in A-C), Hnt (red in D-E), Cut (red in F-H) or β-gal (red in I-J). **(A-C) **In the wild type egg chamber at stage 9, STAT92E accumulates to high levels in nuclei at the posterior pole, with gradual reduction toward the center of the chamber (A, A'). STAT92E nuclear accumulation was present only in the outer layer of the multilayered *scrib*^2 ^(B-B") and *dlg*^*m*52 ^(C-C") clones. Note that the wild type polar cells (arrows in B, B', C and C') are in close proximity to the single layer of outer cells of multilayered clones. **(D, E) **Hnt is expressed in all wild type FCs after stage 6 (D). In *scrib*^2 ^multilayered clones, Hnt expression can be detected in the inner cells, rather in outer layer (E-E"). **(F-H) **In the wild type, expression of Cut is present in FCs until stage 6 (F). In a stage 9 egg chamber with *scrib*^2 ^(G-G") or *dlg*^*m*52 ^(H-H") clone, Cut expression is evident in the outer layer of the multilayered clone. Note that Cut remains in a low level in the inner cells. **(I, J) **The Notch signaling reporter *m7-lacZ *can be activated in all FCs from stage 6-8 in the wild type (I). The *m7-lacZ *activity is localized to the inner cells of multilayered clones in *dlg*^*m*52 ^egg chamber at stage 7 (J-J").

In addition to EGFR pathway, JAK/STAT and Notch pathways are also essential for specification and differentiation of the PFCs. Given that disruption of JAK and/or Notch signaling could cause failure of the FCs at the posterior to adopt a terminal fate, we reckoned that both JAK/STAT and Notch pathways may be also implicated in defective posterior patterning of the epithelium in *scrib/dlg *mutants. To address this question, we investigated whether the above two signaling pathways are properly activated in *scrib *or *dlg *mutant clone cells at the posterior. In the case of JAK/STAT pathway, the nuclear accumulation of STAT92E protein is considered as an indicator of the signaling activation (Fig 4A) [6,27,28,43]. We, therefore, assessed JAK signaling activity in the mutant clones by analyzing the subcellular localization of STAT92E protein. In our experiments, STAT92E nuclear accumulation was present in outer layer, but not in inner cells of the multilayered clones (*scrib*^2^, n = 32; *scrib*^1^, n = 12; *dlg*^*m*52^, n = 20) (Fig 4B, C and data not shown), indicating that JAK/STAT pathway can still be activated in part of the posterior mutant cells. To explain why the mutant FCs at the posterior hardly express the terminal fate markers, we need to further determine how mutations in *scrib/dlg *affect Notch signaling. For this purpose, we next checked the expression pattern of Hindsight (Hnt) and Cut, the target genes of Notch signaling in the mutant clones at the posterior. In the wild type, the expression of Hnt is induced by Notch pathway, whereas this signaling controls downregulation of Cut [44,45] (Fig 4D, F). Significantly, loss of Notch signaling activity is evident in outer layer of all posterior multilayered clones mutant for *scrib *or *dlg*, as indicated by absence of Hnt (*scrib*^2^, n = 51; *scrib*^1^, n = 11; *dlg*^*m*52^, n = 21) (Fig 4E and data not shown) or prolonged expression of Cut (*scrib*^2^, n = 30; *scrib*^1^, n = 9; *dlg*^*m*52^, n = 23) (Fig 4G, H and data not shown). In contrast, we found that Notch pathway is activated in inner cells of the clones in a variable percentage of *scrib/dlg *mosaic chambers (Hnt: *scrib*^2^, 92.2%, n = 51; *scrib*^1^, 90.9%, n = 11; *dlg*^*m*52^, 57.2%, n = 21; Cut: *scrib*^2^, 90%, n = 30; *scrib*^1^, 88.9%, n = 9; *dlg*^*m*52^, 69.6%, n = 23) (Fig 4E, G, H and data not shown). Disruption of Notch signaling in outer cells of the multilayered clones was confirmed by using a Notch-dependent transcriptional reporter, *m7-lacZ *[24,46]. As shown in Fig 4J, in all *dlg*^*m*52 ^clones tested in this report, *m7-lacZ *expression was beyond detection in the outer layer of cells, whereas the inner cells in contact with the germline express this reporter (*dlg*^*m*52^, 100%, n = 22). Altogether, the data strongly suggest that loss of *scrib/dlg *function results in disruption of JAK signaling in inner cells of multilayered clones, and aberrant Notch pathway in outer layer of cells. Thus, JAK and Notch signaling can not be coordinately activated in the mutant FCs at the posterior. These defects in the coordinated signaling activities could underlie the observations that *scrib/dlg *mutant FCs at the posterior domain barely adopt a default terminal fate.

In sum, loss-of-function mutations in *scrib/dlg *cause perturbation of coordinated activities of the three signaling pathways, which could well explain why the mutant FCs at the posterior lose the PFC fate and do not adopt an AFC fate either.

### Mutations in *scrib/dlg *cause aberrant anterior patterning of the follicular epithelium

**Figure 5:**
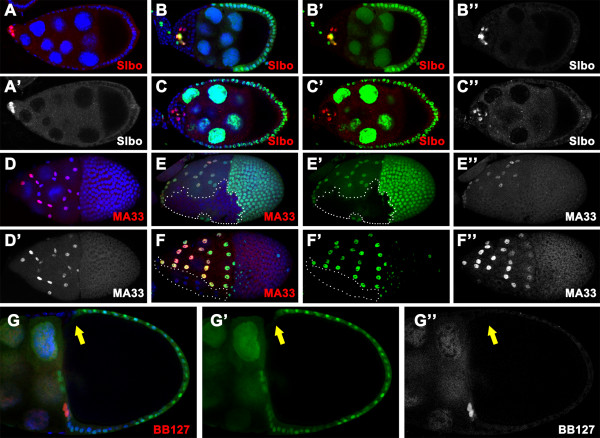
**Inactivation of *scrib *and *dlg *cause aberrant anterior patterning of the follicular epithelium**. Wild type (A and D) and *scrib*^2 ^(B, E and G) or *dlg*^*m*52 ^(C and F) mosaic egg chambers labeled by the absent of the nuclear GFP (green), stained for nuclei (DAPI, blue) and Slbo (red in A-C) or β-gal (red in D-G). **(A-C) **In the *scrib*^2 ^(B-B") or *dlg*^*m*52 ^(C-C") multilayered clones at the anterior of stage 9 egg chambers, a number of FCs located around the polar cells can express Slbo and adopt the border cell fate. **(D-F) **In the wild type, enhancer trap reporter MA33 specifically labels the stretched cell population, which forms a squamous epithelium over the nurse cells through morphogenesis during stage 9-10 (D, D'). No MA33 expression is found in the mutant cells of *scrib*^2 ^(E, E") or *dlg*^*m*52 ^(F, F") stretched cell clone (outlined with white dots). Note that the mutant cells do not flatten and migrate towards covering the nurse cells (E, E'). **(G) **Compared with the stage 10b wild type egg chamber (Fig 3E, E'), clones homozygous for *scrib*^2 ^neither express the centripetal cell specific marker BB127 nor migrate in between the oocyte and nurse cells (arrow in G-G").

**Figure 6:**
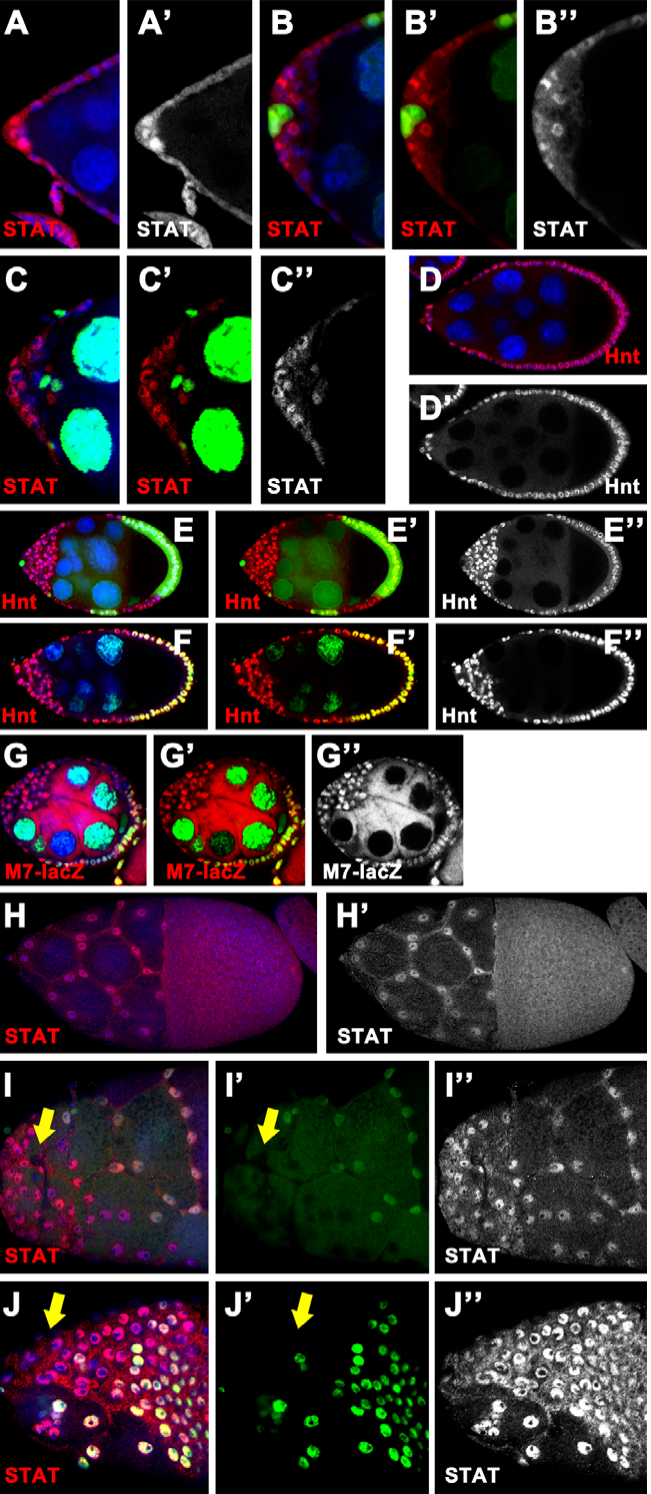
**JAK and Notch signaling can be activated in *scrib/dlg *mutant FCs at the anterior**. Wild type (A, D and H) and *scrib*^2 ^(B, E and I) or *dlg*^*m*52 ^(C, F, G and J) mosaic egg chambers labeled by the absent of the nuclear GFP (green), stained for nuclei (DAPI, blue) and STAT92E (red in A-C and H-J), Hnt (red in D-F) or β-gal (red in G). **(A-C) **STAT92E protein accumulates in the nuclei predominantly at the anterior pole of the stage 8 wild type egg chamber (A, A'). This nuclear accumulation can still be observed in a number of anterior clone cells surrounding polar cells in *scrib*^2 ^(B-B") or *dlg*^*m*52 ^(C-C") mosaic chambers at stage 8. **(D-F) **At stage 9 Hnt is expressed in all FCs in the wild type egg chamber (D, D'). Staining of Hnt is also evident in almost all mutant cells of the *scrib*^2 ^(E-E") or *dlg*^*m*52 ^(F-F") multilayered clone. **(G) **In stage 6 egg chamber with *dlg*^*m*52 ^clone at the anterior, *m7-lacZ *reporter is expressed in all mutant AFCs (G-G"). **(H-J) **In stage 10 wild type egg chamber, STAT92E nuclear accumulation is observed in the stretched cells (H, H'). STAT92E protein can accumulate in the nuclei of *scrib*^2 ^(I, I") or *dlg*^*m*52^(J, J") clone cells at the anterior to a level comparable with that in the wild type stretched cells. Note that the mutant cells can not flatten and migrate normally (arrows in I, I', J and J').

The graded activities of JAK signaling regulate subdivision of the AFCs into three distinct cell types along the AP axis [6,47]. Differentiation of the three AFC subpopulations also requires proper Notch signaling, as it occurs in the mainbody and PFC cells. We showed that loss of *scrib/dlg *function causes defects in coordinated activities of EGFR, JAK/STAT and Notch pathways in FCs at the posterior, which are causally linked to loss of the PFC fate. This finding prompted us to further investigate how the anterior of the follicular epithelium is patterned in *scrib/dlg *mutant chambers. To this end, we generated the mutant clones at the anterior, and first examined the mosaic chambers for expression of either molecular marker or enhancer trap lines that label specific AFC fates. In an overwhelmingly high percentage of stage 8/9 chambers with *scrib *or *dlg *mutant clones covering the entire anterior domain (*scrib*^2^, 95.5%, n = 67; *dlg*^*m*52^, 86.3%, n = 51), Slbo was expressed in a number of FCs located around the polar cells, predominantly in outer layer of the multilayered clone cells (Fig 5B, C) (Table 2). This data suggest that induction of border cells, the most terminal anterior fate, still occur in *scrib/dlg *mutants. Consistent with the previous report [48], we observed that the border cell migration in stage 10 *scrib *mutant chambers was completely blocked (data not shown). Moreover, a similar phenotype of border cell migration was also detected in *dlg *mutant chambers at stage 10 (data not shown). To understand the mechanisms underlying expression of the border cell marker at the most anterior terminal of *scrib/dlg *mutant chambers, we further analyzed the coordinated activities of JAK and Notch signaling. In the case of JAK/STAT pathway, STAT92E nuclear accumulation was evident in the mutant FCs surrounding polar cells at the anterior pole (*scrib*^2^, 100%, n = 15; *dlg*^*m*52^, 100%, n = 14) (Fig 6B, C), showing the presence of JAK signaling activity. In parallel, activation of Notch signaling occurs in each anterior FCs of most of mosaic chambers tested, as indicated by the expression of Hnt and *m7-lacZ *in the mutant cells (Hnt: *scrib*^2^, 93.7%, n = 47; *dlg*^*m*52^, 87.7%, n = 57; *m7-lacZ*: *dlg*^*m*52^, 52.2%, n = 46) (Fig 6E, F, and 6G). Thus combined JAK and Notch signaling activity pattern provides a good basis that a number of the most anterior cells in *scrib/dlg *mutant follicular epithelium can still adopt a border cell fate.

**Table 2:** Induction of distinct AFC fate in *scrib/dlg *mutant clone cells at the anterior terminal

	Absence of The AFC Fate in AFC Clones	
	
AFC Markers	*scrib* ^2^	*dlg* ^*m*52^
Slbo	4.5% (n = 67)	13.7% (n = 51)
MA33	26.5% (n = 34)	27.6% (n = 29)
BB127	77.8% (n = 27)	ND

We next examined effects of *scrib/dlg *mutations in specification of the more central anterior fates, stretched and centripetal cells populations using enhancer trap line MA33 and BB127 respectively. As depicted in Fig 5E, F and Table 2, in a certain percentage of stage 10 *scrib *or *dlg *mutant egg chambers, MA33 expression was absent in clones at the stretched cell territory (*scrib*^2^, 26.5%, n = 34; *dlg*^*m*52^, 27.6%, n = 29), implying a defect in stretched cell differentiation. Starting from stage 9 specified stretched cells undergo a morphogenetic change to become a squamous epithelium covering nurse cells at the anterior [2]. It is conceivable that defective patterning of the stretched cell population will subsequently block its spreading, which happens during the morphogenesis. As predicted, in vast majority of *scrib/dlg *mutant clones with defective differentiation of the stretched cells, the mutant cells failed to spread out and adopt the squamous morphology indicative of aberrant morphogenesis (*scrib*^2^, 88.8%, n = 9; *dlg*^*m*52^, 87.5%, n = 8) (Fig 5E). Likewise, loss of *scrib *function in FCs at the anterior resulted in a failure to express BB127 in a high percentage of stage 10b mosaic chambers harboring both stretched and centripetal cell clones (77.8%, n = 27) (Fig 5G and Table 2), indicating a loss of centripetal cell fate. Further study revealed that the defective "centripetal cells" can not migrate centripetally (90.4%, n = 21) (Fig 5G). Thus, we conclude that mutations in *scrib/dlg *perturb specification of the more central anterior fates. Given that the graded activities of JAK/STAT pathway are necessary for determining specific fates within the anterior terminal domain, we sought to analyze JAK signaling activity in clones harboring the stretched and centripetal cell populations by examining the nuclear accumulation of STAT92E protein. In the wild type, nuclear accumulation of STAT92E indicative of the JAK signaling activities is still present in the specified squamous FCs covering the nurse cells at stage 10 (Fig 6H), but absent in specified centripetal cells (date not shown). We, therefore, chose to determine whether mutations in *scrib/dlg *perturb JAK signaling during patterning and early morphogenesis of the stretched cell population. Strikingly, in all defective "stretched cells" of *scrib/dlg *mutant clones (*scrib*^2^, n = 25; *dlg*^*m*52^, n = 21), STAT92E protein accumulates in nuclei to a level comparable with that in wild type stretched cells (Fig 6I, J), indicating that activation of JAK/STAT pathway can still occur in the mutant cells. Thus, these data suggest that defective patterning of the stretched cell population induced by loss of *scrib *or *dlg *does not require disruption of the JAK/STAT pathway.

Overall, mutations in *scrib/dlg *cause an aberrant anterior patterning of the follicular epithelium, particularly a defect in specification of the stretched and centripetal cell fates. In this circumstance, defective AFC cell fate induction is closely correlated with the aberrant morphogenesis.

### scrib genetically interacts with *dlg *in posterior patterning of the epithelium

**Figure 7:**
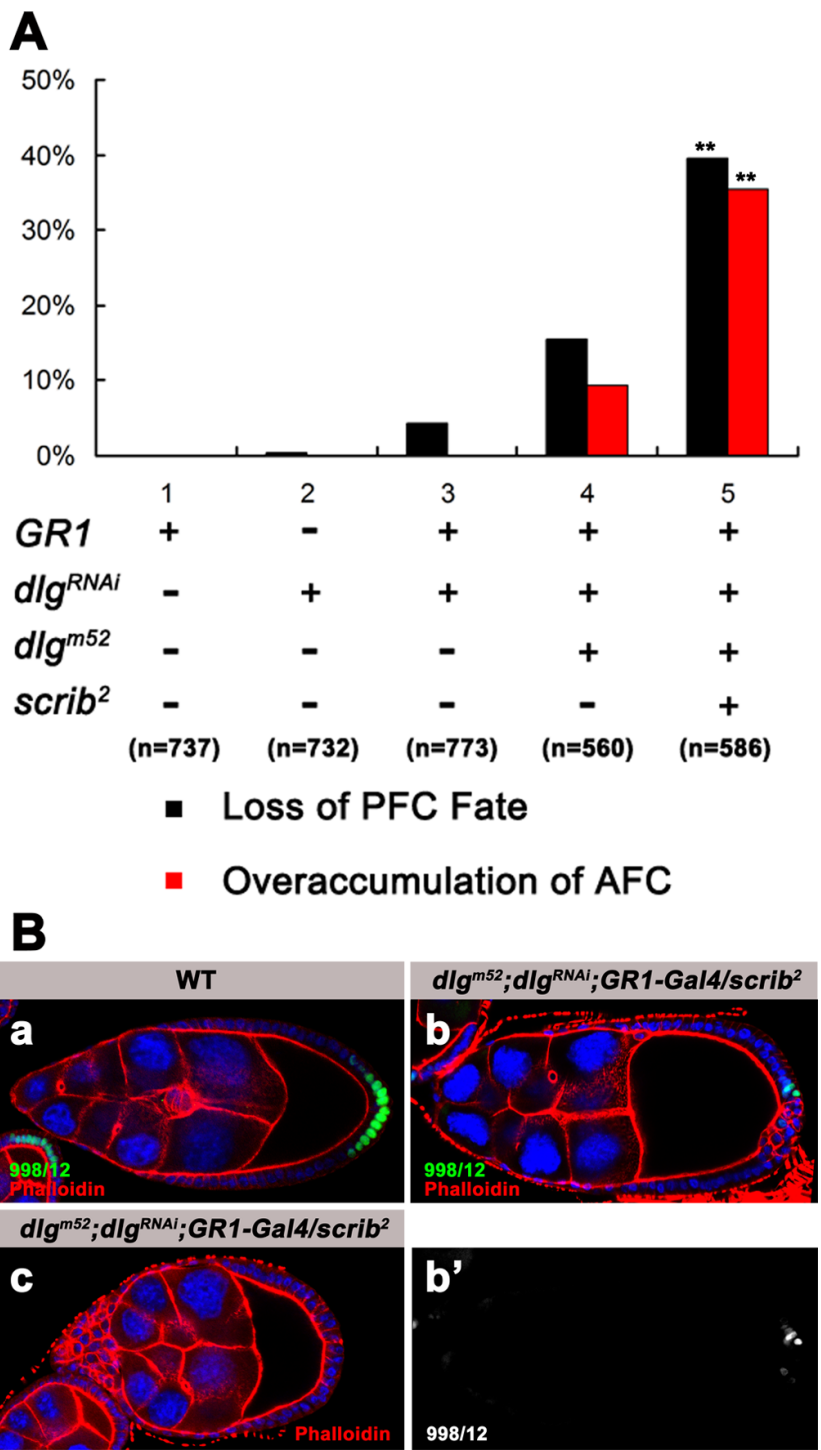
***scrib *shows genetic interaction with *dlg *in posterior patterning of the follicular epithelium**. **(A) **Quantification of loss of the PFC fate and FC overaccumulation at the anterior. Heterozygous *scrib*^2 ^increases the penetrance of loss of the PFC fate and overaccumulation of the AFCs in *dlg*^*m*52^/+; *dlg*^*RNAi *^egg chambers in which *GR1-Gal4*-driven expression of the *dlg*^*RNAi *^transgene is induced in all FCs heterozygous for *dlg*^*m*52^(** *P *< 0.01). **(B) **Egg chambers at stage 9 from wild type (a) and *dlg*^*m*52^/+; *dlg*^*RNAi*^; *GR1-Gal4/scrib*^2 ^females (b, c) stained for β-gal (green in a, b; white in b'), nuclei (DAPI, blue), and Phalloidin (red in a, b and c). The specified PFCs can be visualized by the enhancer trap marker 998/12 in stage 9 wild type egg chamber (a). Loss of 998/12 expression is evident in most of the multilayered FCs at the posterior in *dlg*^*m*52^*/+; dlg*^*RNAi*^; *GR1-Gal4/scrib*^2 ^egg chambers in which endogenous *dlg *is knockdowned in all FCs doubly heterozygous for *dlg*^*m*52 ^and *scrib*^2 ^(b, b'). In these mutant chambers, FC overaccumulation is also observed at the anterior (c).

A genetic interaction between *scrib *and *dlg *in controlling migration and invasion of FCs has been reported in a recent study [48]. Considering that *scrib *and *dlg *have a very similar mutant phenotype of defective AFC and PFC fate induction at oogenesis, we tested for genetic interactions between the two genes in AP patterning of the epithelium. In this experiment, an RNAi transgene of *dlg*, *UAS-dlg*^*RNAi *^available from the VDRC, was employed to specifically knockdown expression of *dlg *in the epithelium. Prior to test for the genetic interactions, we validated the specificity of this transgene in down-regulation of the endogenous *dlg *expression. First, we targeted expression of the transgene in posterior compartments of the wing imaginal discs by using *en-Gal4 *driver and examined *dlg *expression in the epithelial cells. The immuno-staining assay clearly showed a remarkable reduction of Dlg in those cells (data not shown). Second, we knocked down *dlg *in all FCs heterozygous for *dlg*^*m*52 ^for phenotypic analysis. As depicted in Fig 7A, the *dlg*^*RNAi*^-knockdown induced phenotype was enhanced by expression of this RNAi transgene in *dlg*^*m*52 ^heterozygosity, as indicated in loss of the PFC fate and presence of multilayered FCs at the anterior. Collectively, these data demonstrated that this RNAi transgene can specifically target the endogenous *dlg *for an inactivation.

We next investigated whether *scrib *interacts genetically with *dlg *in subdivision of the follicular epithelium into distinct cell types. *GR1-Gal4*-driven expression of *dlg*^*RNAi *^in the entire epithelium heterozygous for *dlg*^*m*52 ^caused failure of the FCs at the posterior to differentiate properly in a certain percentage of the mutant chambers (Fig 7A). Strikingly, heterozygosity for *scrib*^2 ^increases the penetrance of defective PFC fate induction in *dlg*^*m*52^*/+; dlg*^*RNAi *^mutant chambers (Fig 7A, Bb and 7Bb'), strongly suggesting that *scrib *and *dlg *act in a common pathway to function in posterior patterning of the epithelium. Likewise, genetic interactions of *scrib *with *dlg *were also observed for FC overaccumulation at the anterior (Fig 7A and 7Bc). In this case, however, development of the mutant chambers with multilayered FCs at the anterior was completely blocked before expression of the enhancer trap markers for the AFC fates appears at stage 10. This impeded a direct assay of the AFC fate in the mutant epithelium with cell overaccumulation at the anterior. For those mutant chambers with the FCs at the anterior remaining a monolayer, we detected expression of the AFC fate marker L53b in anterior mutant FCs (data not shown). Thus, study of genetic interactions between *scrib *and *dlg *in the anterior patterning is inconclusive.

## Discussion

In the present study we show that *scrib/dlg *function in both anterior and posterior patterning of the follicular epithelium. While removal of *scrib *or *dlg *function from the FCs at the posterior terminal completely blocked specification and differentiation of the PFCs, loss-of-function mutations in *scrib/dlg *at the anterior domain resulted in a partially penetrant phenotype of defective AFC cell fate induction as indicative of absence of the stretched and centripetal cell types at stage 10 of egg chambers. The differential regulation of the PFC and AFC cell differentiation by *scrib/dlg *could be attributable to the distinct signaling basis underlying the follicular patterning at the two terminals. In the case of posterior patterning, the combinatorial and sequential activities of JAK/STAT, EGFR and Notch signaling pathways play key roles in this process. The fully penetrant phenotype of aberrant PFC fate specification in posterior *scrib/dlg *mutant clones can be explained by the fact that inactivation of *scrib *or *dlg *completely perturb the EGFR signaling. Further investigation demonstrated that *scrib/dlg *mutation also causes defects in coordinated activation of JAK/STAT and Notch pathways in each multilayered clone cell, as evident in localized activity of JAK signaling and Notch signaling in the outer layer and inner layer respectively (Fig 4B, C, E, G, H, and 4J). Thus, the mutant cells at the posterior terminals generally do not adopt a terminal cell fate either. At the anterior, AFC fate induction requires JAK and Notch signaling activities. Current data, however, do not exclude a possibility that other unknown signals are involved in specifying the distinct AFC cell types, e.g. the stretched and centripetal cells[1,6,7]. We observed that while JAK signaling activity is present in the FCs surrounding the polar cells, preferentially in outer cells of the multilayered clones at the anterior terminals, activation of Notch pathway occurs in almost all mutant cells. The combined pattern of JAK and Notch signaling activity in the anterior mutant cells provides a good basis that the border cell, at least Slbo-expressing cell fate, can be induced in the most anterior region of the multilayered clones. However, our clonal analysis revealed specification of the stretched or centripetal cell types in corresponding mutant clones at the anterior is disrupted, albeit to a lesser extent. Considering the presence of JAK signaling activity in the mutant clone cells, we assume that loss-of-function mutation of *scrib/dlg *may perturb other unidentified signals implicated in patterning of the stretched and centripetal cell subpopulations.

We have identified in this report aberrant EGFR signaling pathway as the mechanism underlying defective PFC fate induction in *scrib/dlg *mutant FCs. In addition to EGFR pathway, loss of *scrib *or *dlg *function at the terminal domains can differentially affect JAK and Notch signaling activities in the multilayered clone cells. JAK signaling was absent in inner cells of the multilayered clones at the two terminals. Conversely, loss of Notch activity was localized to outer cells of the posterior multilayered clones. This discrepancy is likely to be linked to the spatial and temporal control of each signaling activity with respect to growth and patterning of the FC layer during oogenesis. Starting from stage 6 of oogenesis, Notch signaling is activated in all epithelial FCs for inducing the mitotic-to-endocycle transition [12,13]. Given that at this time the multilayered cells have been formed in *scrib/dlg *mutant clones at the terminals, it can be imagined that activation of Notch pathway occurs only in the mutant cells directly contacting the Delta-producing germ cell, as shown in the inner cells of the posterior mutant clones (Fig 4E, G, H and 4J). This scenario was further justified by the recent report that loss of *lgl *causes the same aberrant Notch signaling pattern in the posterior multilayered FCs as *scrib/dlg *mutation does [49]. Surprisingly, the multilayered *scrib/dlg *mutant clones at the anterior display a distinct Notch signaling pattern in which almost all FCs regardless of their spatial relation with the oocyte are positive for Notch activation (Fig 6E, F and 6G). Although at this point we do not understand the basis for this controversial pattern of Notch activity in the mutant clones at different terminals, this mechanism may partly underlie the observation in the present study that loss-of-function mutations in *scrib/dlg *differentially affect anterior and posterior patterning of the epithelium.

Likewise, the distinct pattern for JAK/STAT pathway activity between the inner and outer cells in *scrib/dlg *mutant clones is probably due to the spatial location of the ligand sources relative to the multilayered cells. Indeed, analysis of the polar cell positioning in the mutant clones revealed that the polar cells are in close proximity to the single layer of outer cells that retain JAK signaling activity (*scrib*^2^, 95.3%, n = 43; *dlg*^*m*52^, 88.9%, n = 27) (see Additional file 1) (Fig 4B, C). The positioning of the polar cells led us to argue that inner cells in the multilayered clones do not respond to the signaling ligand Unpaired secreted from polar cells, presumably due to their spatial relation with the ligand source. However, unlike the Notch pathway, JAK signaling is activated in FCs at the terminal domains of the egg chambers at early oogenesis after the polar/stalk cells are specified [6,7,22]. This temporal regulation might be alternatively responsible for the distinct JAK/STAT activity pattern in the multilayered clone cells. In this model, we assume that JAK/STAT pathway is activated in the mutant FCs at the terminal domains prior to occurrence of the cell overaccumulation. Thus, the presence of JAK signaling activity in single outer cell layer of the multilayered *scrib/dlg *mutant clones may indicate the initial activation of JAK/STAT pathway induced by the polar cells for specifying the terminal fate at early oogenesis. On the contrary, the inner cells deriving from the overaccumulation fail to respond to Unpaired ligand. Further studies in this direction will better define the underlying mechanisms for defective follicular patterning elicited by loss of *scrib *or *dlg*.

The phenotypic effects of *scrib/dlg *mutation in posterior patterning of the epithelium are similar to those of the Hpo pathway deficiency [24-26]. Further characterization of the patterning defects, however, reveals distinct underlying mechanisms for these two instances. In the case of the Hpo pathway, loss of the pathway component Hippo, Salvador, or Warts disrupts Notch signaling in all mutant FCs at the posterior via interfering with endocytosis of the Notch receptor, thereby resulting in aberrant PFC cell specification and differentiation at mid-oogenesis [24-26]. By contrast, activation of the Notch signaling is evident in inner cells of the multilayered *scrib/dlg *mutant clones at the posterior (Fig 4E, G, H and 4J) and almost all the multilayered clone cells at the anterior (Fig 6E, F and 6G). Furthermore, the fully disrupted EGFR pathway associated with the posterior patterning defects conferred by loss of *scrib *or *dlg *clearly distinguishes the Hpo pathway from *scrib/dlg *in the signaling basis for the mutant phenotype of defective PFC fate induction ([24-26] and this paper). In *Drosophila*, *scrib/dlg *are known to encode scaffolding proteins that are localized at the septate (basolateral) junctions of epithelial cells, and regulate the apico-basal cell polarity[31,35,50-52]. Previous studies have demonstrated that mutations in *scrib/dlg *disrupt the epithelial polarity in the FCs at the terminal domains of egg chambers, exhibiting mislocalized cell polarity proteins [31,48,53]. Based on this fact, one would surmise that the polarity defects observed in the posterior *scrib/dlg *mutant FCs perturb the apical accumulation of EGFR receptors, rendering these cells incompetent to respond to EGF signals due to failure of EGFR activation. However, further studies in this paper disapproved this simple scenario. Instead, we found that ERK is di-phosphorylated in the posterior FCs lacking *scrib *or *dlg*, suggesting that the EGFR in the mutant cells can still be activated in response to Grk signal. Thus, this finding points out that blocking in signal transduction from the activated ERK to the downstream targets elicited by loss of *scrib *or *dlg *may result in failure of the mutant FCs at the posterior to respond to EGFR signals.

Once the specified FC cell types are induced, each cell population will undergo a unique morphogenetic change and execute respective functions [2]. Remarkably, we observed a concurrent defect in morphogenesis of those anterior *scrib/dlg *mutant clone cells with aberrant patterning of the stretched or centripetal cell subpopulation (Fig 5E, G and Fig 6I, J). It would be interesting and important to determine whether *scrib/dlg *is implicated in morphogenesis of the patterned follicular epithelium as well. For this purpose, we need to identify a *scrib *or *dlg *mutant allele for certain genetic background in which the morphogenesis can be uncoupled from the patterning process. Under such circumstance could we generate *scrib/dlg *mutant clones with proper patterning of the AFC cell types, and then test how the subsequent morphogenesis occurs in the specified cell subpopulations, e.g. the stretched and centripetal cells. Likewise, a hypomorphic *scrib *or *dlg *allele with certain reduced activity could be of great value to understanding better how the specified PFCs function in polarization of the oocyte at mid-oogenesis. Interestingly, we found that RNAi-mediated knockdown of the endogenous *dlg *expression alone in the follicular epithelium can disrupt the oocyte polarity as indicative of mislocalization of Stau, but properly induce the PFC fate (our unpublished data). This unexpected observation led us to propose that expression of this *dlg*^*RNAi *^transgene may specifically perturb the process in which the specified PFCs control formation of the oocyte polarity. Thus, screening a *Drosophila *mutant library such as the transgenic RNAi library for gene(s) modifying the *dlg*^*RNAi *^phenotype would unveil the mechanisms responsible for involvements of *dlg *in regulation of the PFC function.

## Conclusion

In this paper we present the first demonstration that the tumor suppressor genes *scrib *and *dlg *are required in the FCs for patterning of the follicular epithelium along the AP axis during *Drosophila *oogenesis. Genetic interaction of *scrib *with *dlg *in specification and differentiation of PFCs indicates a cooperative role between these two genes. While the data clearly show a differential role of *scrib/dlg *in anterior and posterior patterning of this epithelial layer, the underlying mechanisms await further investigations. Overall, study in this direction may provide alternatives for addressing *scrib/dlg*-mediated regulation of cell polarity and proliferation in epithelial tissues.

## Methods

### Fly stocks and genetics

All *Drosophila *stocks were maintained and crossed at 25°C according to standard procedures. Egg chamber stages are according to Spradling [54]. The *Canton S (CS) *strain was used as wild type. *scrib*^2 ^and *scrib*^1 ^are null alleles of *scrib *[31,52], and *dlg*^*m*52 ^is a null allele of *dlg *[50]. The transgenic RNAi line for *dlg*, *UAS-dlg*^*RNAi*^, was obtained from Vienna *Drosophila *RNAi Centre (VDRC, Transformant ID 41134). *en-Gal4 *(gift from A Bergmann) [55] and *GR1-Gal4 *(gift from T Schüpbach) [56] were used to drive its expression.

Mutant clones were generated by mitotic recombination using FLP/FRT Technique [57]. Homozygous *scrib*^2^, *scrib*^1 ^or *dlg*^*m*52 ^clones were generated by crossing *FRT82B scrib*^2^*/TM3 Sb *(gift from D Bilder) or *FRT82B scrib*^1^*/TM3 Ser *(gift from HE Richardson) to *yw hsFLP;FRT82B ubi-GFPnls*, or crossing *FRT101 dlg*^*m*52^*/FM7 *(gift from S Goode) to *FRT101 hGFP/FM7; hsFLP/CyO *(gift from DA Harrison) or *FRT101 tub-lacZ hsFLP/FM7 *(gift from S Goode). To obtain follicle cell clones, the flies were heat-shocked as 3rd instar larvae and pupae at 37°C for 1 h on 4 consecutive days. Before dissection, all adults were put into fresh food vials for 2 days. The following enhancer trap markers were incorporated into the above fly strains for making *scrib*^2 ^or *dlg*^*m*52 ^clones: 998/12 (gift from D St Johnston) [5], *Kinesin-lacZ *(*yw Kinesin-lacZ*, gift from D St Johnston and KZ503, gift from YN Jan) [17], *kek *enhancer trap line BB142 (gift from T Schüpbach) [23,37], *m7-lacZ *(gift from T Xie) [24,46], MA33, BB127, L53b (gift from DA Harrison) [8,41], *dpp-lacZ *(Bloomington *Drosophila *Stock Center) [42]. 998/12 was recombined onto the *FRT82B, scrib*^2 ^chromosome using meiotic recombination.

### Antibodies and immunofluorescence

For antibody staining, ovaries were dissected into phosphate buffered saline (PBS) with 0.1% bovine serum albumin, fixed in 4% paraformaldehyde for 30 min and washed three times with PBST (0.3% Triton X-100 in PBS) except for anti-STAT92E and anti-dp-ERK staining. Then ovaries were permeabilized in PBS with 1% Triton X-100 for 1 h at room temperature (RT) followed by a 2 h incubation in PBST with 10% normal goat serum. Primary antibodies were incubated with ovaries at 4°C overnight. On the following day, ovaries were washed with PBST three times for 20 min and blocked for 1 h at RT. Then they were incubated with secondary antibodies or Phalloidin-TRITC (Sigma) at RT for 2 h, and stained with DAPI (Molecular Probes) for 10 min. Finally, ovaries were rinsed three times with PBST and mounted in VECTASHIELD Mounting Medium (Vector Laboratories). For anti-STAT92E staining ovaries were washed and incubated in PBS with 0.3% Tween-20. For anti-dp-ERK staining ovaries were fixed for 30 min in 8% formaldehyde, rinsed for an hour in PBS with 0.1% Tween-20, and stored overnight in methanol. After progressive rehydration and block, the ovaries were incubated with anti-dp-ERK antibodies.

The following primary antibodies were used in this work: rabbit anti-Stau (1:2000 gift from D St Johnston) [58], mouse anti-β-gal (1:10 DSHB 40-1a), rabbit anti-β-gal (1:50000 Cappel), rabbit anti-DG (1:3000 gift from WM Deng) [59], mouse anti-dp-ERK (1:200 Cell Signaling) [39,40], rabbit anti-STAT92E (1:1000 gift from SX Hou) [60], mouse anti-Hnt (1:200 DSHB 1G9), mouse anti-Cut (1:100 DSHB 2B10), rat anti-Slbo (1:500 gift from P Rørth) [61], mouse anti-Dlg (1:1000 DSHB 4F3), mouse anti-Fas3 (1:200 DSHB 7G10). Secondary antibodies conjugated with Alexa Fluor 488, 546, 568 (Molecular Probes) were used at 1:1000 dilutions.

Confocal images were captured on Zeiss LSM 510 META laser scanning microscope and processed in Adobe Photoshop.

### Statistical analysis

*P*-values were calculated by applying χ^2^-test.

## Authors' contributions

QL designed experiments, performed the fly genetics and immunofluorescence, and participated in drafting the manuscript. LS performed the experimental work in *dlg*. TX carried out the fly genetics and participated in drafting the manuscript. WX participated in the fly genetics and immunofluorescence. WC participated in the experimental work in *dlg*. YG participated in the experimental work in *scrib*. MZ provided continuous intellectual input and interpretation of data. LY performed the fly genetics and clonal analysis. ML conceived, coordinated the project, and wrote the manuscript. All authors read and approved the final manuscript.

## Supplementary Material

Additional file 1 **The positioning of wild type polar cells in the outer layer of *scrib/dlg *mutant clone FCs**. Strikingly, the pairs of wild type polar cells, marked by Fas3 (arrows in B, B', C and C') are in close proximity to the outer layer of multilayered *scrib*^2 ^(B, B') or *dlg*^*m*52 ^(C, C') clones at the posterior. Click here for file

## References

[B1] Horne-Badovinac S Bilder D Mass transit: epithelial morphogenesis in the *Drosophila *egg chamber Dev Dyn 2005 232 559 574 10.1002/dvdy.20286 15704134

[B2] Wu X Tanwar PS Raftery LA *Drosophila *follicle cells: morphogenesis in an eggshell Semin Cell Dev Biol 2008 19 271 282 10.1016/j.semcdb.2008.01.004 18304845 PMC2430523

[B3] Poulton JS Deng WM Cell-cell communication and axis specification in the *Drosophila *oocyte Dev Biol 2007 311 1 10 10.1016/j.ydbio.2007.08.030 17884037 PMC2174919

[B4] Huynh JR St Johnston D The origin of asymmetry: early polarisation of the *Drosophila *germline cyst and oocyte Curr Biol 2004 14 R438 449 10.1016/j.cub.2004.05.040 15182695

[B5] Gonzalez-Reyes A St Johnston D Patterning of the follicle cell epithelium along the anterior-posterior axis during *Drosophila *oogenesis Development 1998 125 2837 2846 9655806 10.1242/dev.125.15.2837

[B6] Xi R McGregor JR Harrison DA A gradient of JAK pathway activity patterns the anterior-posterior axis of the follicular epithelium Dev Cell 2003 4 167 177 10.1016/S1534-5807(02)00412-4 12586061

[B7] Denef N Schupbach T Patterning: JAK-STAT signalling in the *Drosophila *follicular epithelium Curr Biol 2003 13 R388 390 10.1016/S0960-9822(03)00317-8 12747848

[B8] Roth S Neuman-Silberberg FS Barcelo G Schupbach T *cornichon *and the EGF receptor signaling process are necessary for both anterior-posterior and dorsal-ventral pattern formation in *Drosophila* Cell 1995 81 967 978 10.1016/0092-8674(95)90016-0 7540118

[B9] Gonzalez-Reyes A Elliott H St Johnston D Polarization of both major body axes in *Drosophila *by *gurken-torpedo *signalling Nature 1995 375 654 658 10.1038/375654a0 7791898

[B10] Van Buskirk C Schupbach T Versatility in signalling: Multiple responses to EGF receptor activation during Drosophila oogenesis Trends Cell Biol 1999 9 1 4 10.1016/S0962-8924(98)01413-5 10087609

[B11] Ray RP Schupbach T Intercellular signaling and the polarization of body axes during *Drosophila *oogenesis Genes Dev 1996 10 1711 1723 10.1101/gad.10.14.1711 8698232

[B12] Lopez-Schier H St Johnston D Delta signaling from the germ line controls the proliferation and differentiation of the somatic follicle cells during *Drosophila *oogenesis Genes Dev 2001 15 1393 1405 10.1101/gad.200901 11390359 PMC312703

[B13] Deng WM Althauser C Ruohola-Baker H Notch-Delta signaling induces a transition from mitotic cell cycle to endocycle in *Drosophila *follicle cells Development 2001 128 4737 4746 11731454 10.1242/dev.128.23.4737

[B14] Riechmann V Ephrussi A Axis formation during *Drosophila *oogenesis Curr Opin Genet Dev 2001 11 374 383 10.1016/S0959-437X(00)00207-0 11448623

[B15] Steinhauer J Kalderon D Microtubule polarity and axis formation in the *Drosophila *oocyte Dev Dyn 2006 235 1455 1468 10.1002/dvdy.20770 16586443

[B16] St Johnston D Moving messages: the intracellular localization of mRNAs Nat Rev Mol Cell Biol 2005 6 363 375 10.1038/nrm1643 15852043

[B17] Clark I Giniger E Ruohola-Baker H Jan LY Jan YN Transient posterior localization of a kinesin fusion protein reflects anteroposterior polarity of the *Drosophila *oocyte Curr Biol 1994 4 289 300 10.1016/S0960-9822(00)00068-3 7922338

[B18] Januschke J Gervais L Gillet L Keryer G Bornens M Guichet A The centrosome-nucleus complex and microtubule organization in the *Drosophila *oocyte Development 2006 133 129 139 10.1242/dev.02179 16319114

[B19] Schupbach T Germ line and soma cooperate during oogenesis to establish the dorsoventral pattern of egg shell and embryo in *Drosophila melanogaster* Cell 1987 49 699 707 10.1016/0092-8674(87)90546-0 3107840

[B20] Neuman-Silberberg FS Schupbach T The *Drosophila *dorsoventral patterning gene *gurken *produces a dorsally localized RNA and encodes a TGF alpha-like protein Cell 1993 75 165 174 10.1016/0092-8674(93)90688-M 7691414

[B21] Nilson LA Schupbach T EGF receptor signaling in *Drosophila *oogenesis Curr Top Dev Biol 1999 44 203 243 10.1016/S0070-2153(08)60471-8 9891881

[B22] McGregor JR Xi R Harrison DA JAK signaling is somatically required for follicle cell differentiation in Drosophila Development 2002 129 705 717 11830571 10.1242/dev.129.3.705

[B23] Pai LM Barcelo G Schupbach T *D-cbl*, a negative regulator of the Egfr pathway, is required for dorsoventral patterning in *Drosophila *oogenesis Cell 2000 103 51 61 10.1016/S0092-8674(00)00104-5 11051547

[B24] Polesello C Tapon N Salvador-warts-hippo signaling promotes *Drosophila *posterior follicle cell maturation downstream of Notch Curr Biol 2007 17 1864 1870 10.1016/j.cub.2007.09.049 17964162

[B25] Meignin C Alvarez-Garcia I Davis I Palacios IM The salvador-warts-hippo pathway is required for epithelial proliferation and axis specification in *Drosophila* Curr Biol 2007 17 1871 1878 10.1016/j.cub.2007.09.062 17964161 PMC3432442

[B26] Yu J Poulton J Huang YC Deng WM The Hippo pathway promotes Notch signaling in regulation of cell differentiation, proliferation, and oocyte polarity PLoS ONE 2008 3 e1761 10.1371/journal.pone.0001761 18335037 PMC2258146

[B27] Devergne O Ghiglione C Noselli S The endocytic control of JAK/STAT signalling in *Drosophila* J Cell Sci 2007 120 3457 3464 10.1242/jcs.005926 17855388

[B28] Ghiglione C Devergne O Cerezo D Noselli S *Drosophila *RalA is essential for the maintenance of Jak/Stat signalling in ovarian follicles EMBO Rep 2008 9 676 682 10.1038/embor.2008.79 18552769 PMC2475328

[B29] Riechmann V Developmental biology: hippo promotes posterior patterning by preventing proliferation Curr Biol 2007 17 R1006 1008 10.1016/j.cub.2007.10.018 18054759

[B30] Li Q Xin T Chen W Zhu M Li M *Lethal(2)giant larvae *is required in the follicle cells for formation of the initial AP asymmetry and the oocyte polarity during *Drosophila *oogenesis Cell Res 2008 18 372 384 10.1038/cr.2008.25 18268543

[B31] Bilder D Li M Perrimon N Cooperative regulation of cell polarity and growth by *Drosophila *tumor suppressors Science 2000 289 113 116 10.1126/science.289.5476.113 10884224

[B32] Tanentzapf G Tepass U Interactions between the *crumbs*, *lethal giant larvae *and *bazooka *pathways in epithelial polarization Nat Cell Biol 2003 5 46 52 10.1038/ncb896 12510193

[B33] Humbert P Russell S Richardson H Dlg, Scribble and Lgl in cell polarity, cell proliferation and cancer Bioessays 2003 25 542 553 10.1002/bies.10286 12766944

[B34] Bilder D Epithelial polarity and proliferation control: links from the *Drosophila *neoplastic tumor suppressors Genes Dev 2004 18 1909 1925 10.1101/gad.1211604 15314019

[B35] Yamanaka T Ohno S Role of Lgl/Dlg/Scribble in the regulation of epithelial junction, polarity and growth Front Biosci 2008 13 6693 6707 10.2741/3182 18508688

[B36] Keller Larkin M Deng WM Holder K Tworoger M Clegg N Ruohola-Baker H Role of Notch pathway in terminal follicle cell differentiation during *Drosophila *oogenesis Dev Genes Evol 1999 209 301 311 10.1007/s004270050256 11252183

[B37] Schupbach T Roth S Dorsoventral patterning in *Drosophila *oogenesis Curr Opin Genet Dev 1994 4 502 507 10.1016/0959-437X(94)90064-A 7950316

[B38] Poulton JS Deng WM Dystroglycan down-regulation links EGF receptor signaling and anterior-posterior polarity formation in the *Drosophila *oocyte Proc Natl Acad Sci USA 2006 103 12775 12780 10.1073/pnas.0603817103 16908845 PMC1568923

[B39] Gabay L Seger R Shilo BZ In situ activation pattern of Drosophila EGF receptor pathway during development Science 1997 277 1103 1106 10.1126/science.277.5329.1103 9262480

[B40] Guichard A Roark M Ronshaugen M Bier E brother of rhomboid, a rhomboid-related gene expressed during early Drosophila oogenesis, promotes EGF-R/MAPK signaling Dev Biol 2000 226 255 266 10.1006/dbio.2000.9851 11023685

[B41] Fasano L Kerridge S Monitoring positional information during oogenesis in adult *Drosophila* Development 1988 104 245 253 2855515 10.1242/dev.104.2.245

[B42] Twombly V Blackman RK Jin H Graff JM Padgett RW Gelbart WM The TGF-beta signaling pathway is essential for *Drosophila *oogenesis Development 1996 122 1555 1565 8625842 10.1242/dev.122.5.1555

[B43] Silver DL Geisbrecht ER Montell DJ Requirement for JAK/STAT signaling throughout border cell migration in *Drosophila* Development 2005 132 3483 3492 10.1242/dev.01910 16000386

[B44] Sun J Deng WM Notch-dependent downregulation of the homeodomain gene *cut *is required for the mitotic cycle/endocycle switch and cell differentiation in *Drosophila *follicle cells Development 2005 132 4299 4308 10.1242/dev.02015 16141223 PMC3891799

[B45] Sun J Deng WM Hindsight mediates the role of Notch in suppressing Hedgehog signaling and cell proliferation Dev Cell 2007 12 431 442 10.1016/j.devcel.2007.02.003 17336908 PMC1851662

[B46] Assa-Kunik E Torres IL Schejter ED Johnston DS Shilo BZ *Drosophila *follicle cells are patterned by multiple levels of Notch signaling and antagonism between the Notch and JAK/STAT pathways Development 2007 134 1161 1169 10.1242/dev.02800 17332535

[B47] Silver DL Montell DJ Paracrine signaling through the JAK/STAT pathway activates invasive behavior of ovarian epithelial cells in *Drosophila* Cell 2001 107 831 841 10.1016/S0092-8674(01)00607-9 11779460

[B48] Zhao M Szafranski P Hall CA Goode S Basolateral junctions utilize warts signaling to control epithelial-mesenchymal transition and proliferation crucial for migration and invasion of *Drosophila *ovarian epithelial cells Genetics 2008 178 1947 1971 10.1534/genetics.108.086983 18430928 PMC2323789

[B49] Vaccari T Lu H Kanwar R Fortini ME Bilder D Endosomal entry regulates Notch receptor activation in *Drosophila melanogaster* J Cell Biol 2008 180 755 762 10.1083/jcb.200708127 18299346 PMC2265571

[B50] Woods DF Bryant PJ The discs-large tumor suppressor gene of Drosophila encodes a guanylate kinase homolog localized at septate junctions Cell 1991 66 451 464 10.1016/0092-8674(81)90009-X 1651169

[B51] Woods DF Hough C Peel D Callaini G Bryant PJ Dlg protein is required for junction structure, cell polarity, and proliferation control in Drosophila epithelia J Cell Biol 1996 134 1469 1482 10.1083/jcb.134.6.1469 8830775 PMC2120992

[B52] Bilder D Perrimon N Localization of apical epithelial determinants by the basolateral PDZ protein Scribble Nature 2000 403 676 680 10.1038/35001108 10688207

[B53] Goode S Wei J Kishore S Novel spatiotemporal patterns of epithelial tumor invasion in Drosophila discs large egg chambers Dev Dyn 2005 232 855 864 10.1002/dvdy.20336 15712204

[B54] Spradling AC Bate M, Martinez-Arias A Development genetics of oogenesis The Development of Drosophila melanogaster 1993 1 Cold Spring Harbor: Cold Spring Harbor Laboratory Press 1 70

[B55] Xu D Li Y Arcaro M Lackey M Bergmann A The CARD-carrying caspase Dronc is essential for most, but not all, developmental cell death in *Drosophila* Development 2005 132 2125 2134 10.1242/dev.01790 15800001 PMC2519871

[B56] Gupta T Schupbach T Cct1, a phosphatidylcholine biosynthesis enzyme, is required for *Drosophila *oogenesis and ovarian morphogenesis Development 2003 130 6075 6087 10.1242/dev.00817 14597574

[B57] Xu T Rubin GM Analysis of genetic mosaics in developing and adult *Drosophila *tissues Development 1993 117 1223 1237 8404527 10.1242/dev.117.4.1223

[B58] St Johnston D Beuchle D Nusslein-Volhard C *staufen*, a gene required to localize maternal RNAs in the *Drosophila *egg Cell 1991 66 51 63 10.1016/0092-8674(91)90138-O 1712672

[B59] Deng WM Schneider M Frock R Castillejo-Lopez C Baumgartner S Ruohola-Baker H Dystroglycan is required for polarizing the epithelial cells and the oocyte in *Drosophila* Development 2003 130 173 184 10.1242/dev.00199 12441301

[B60] Chen HW Chen X Oh SW Marinissen MJ Gutkind JS Hou SX *mom *identifies a receptor for the *Drosophila *JAK/STAT signal transduction pathway and encodes a protein distantly related to the mammalian cytokine receptor family Genes Dev 2002 16 388 398 10.1101/gad.955202 11825879 PMC155335

[B61] Jekely G Sung HH Luque CM Rorth P Regulators of endocytosis maintain localized receptor tyrosine kinase signaling in guided migration Dev Cell 2005 9 197 207 10.1016/j.devcel.2005.06.004 16054027

