# Supplementary figures and images for: Role of Scrib and Dlg in anterior-posterior patterning of the follicular epithelium during Drosophila oogenesis
Source: BMC Dev Biol. 2009 Dec 1;9:60. doi: 10.1186/1471-213X-9-60 (PMC2810132; doi:10.1186/1471-213X-9-60)

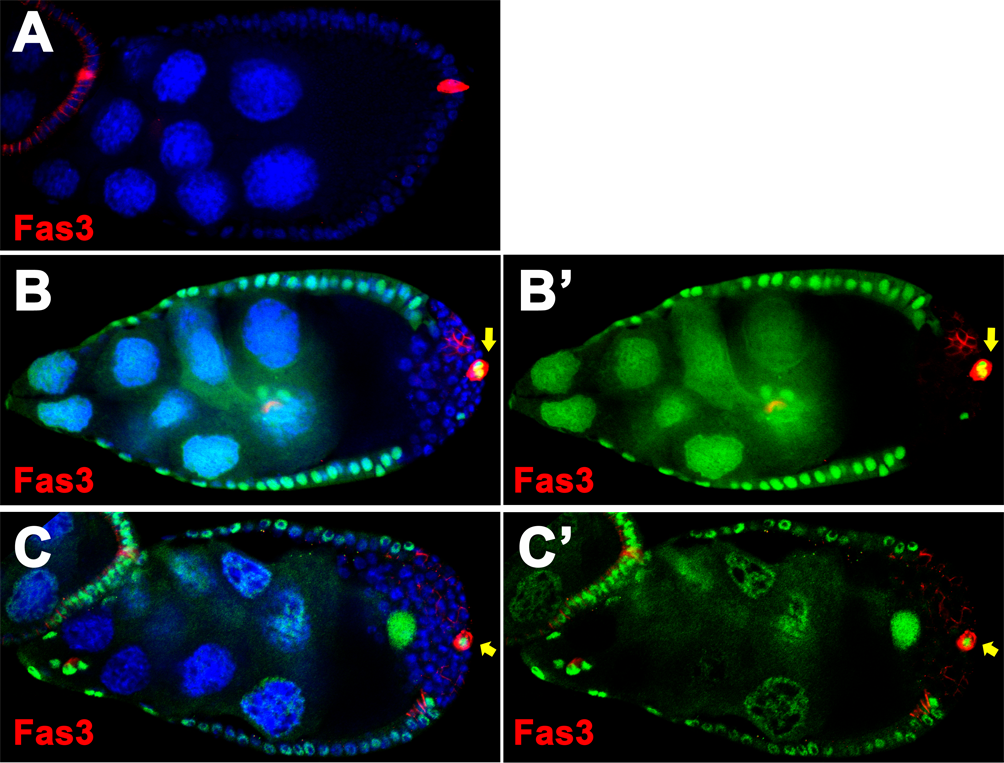

Supplement: Additional file 1 — The positioning of wild type polar cells in the outer layer of scrib/dlg mutant clone FCs. Strikingly, the pairs of wild type polar cells, marked by Fas3 (arrows in B, B', C and C') are in close proximity to the outer layer of multilayered scrib2 (B, B') or dlgm52 (C, C') clones at the posterior. [file 1471-213X-9-60-S1.tiff]
